# Fluorinated Bioactive Glass Nanoparticles: Enamel Demineralization Prevention and Antibacterial Effect of Orthodontic Bonding Resin

**DOI:** 10.3390/ma12111813

**Published:** 2019-06-04

**Authors:** Hyung-Jin Nam, You-Min Kim, Yong Hoon Kwon, Kyung-Hyeon Yoo, Seog-Young Yoon, In-Ryoung Kim, Bong-Soo Park, Woo-Sung Son, Seung-Min Lee, Yong-Il Kim

**Affiliations:** 1 Department of Orthodontics, Dental Research Institute, Pusan National University Dental Hospital, Yangsan 50612, Korea; onnuri00@gmail.com (H.-J.N.); yumini78@naver.com (Y.-M.K.); wsson@pusan.ac.kr (W.-S.S.); 2 Department of Dental Materials, School of Dentistry, Pusan National University, Yangsan 50612, Korea; y0k0916@pusan.ac.kr; 3 School of Materials Science and Engineering, Pusan National University, Busan 46241, Korea; seweet07@pusan.ac.kr (K.-H.Y.); syy3@pusan.ac.kr (S.-Y.Y.); 4 Department of Oral Anatomy, School of Dentistry, Pusan National University, Yangsan 50612, Korea; biowool@pusan.ac.kr (I.-R.K.); parkbs@pusan.ac.kr (B.-S.P.); 5 Institute of Translational Dental Sciences, Pusan National University, Busan 46241, Korea

**Keywords:** anti-demineralization, white spot lesion, bioactive glass, fluorinated bioactive glass

## Abstract

Orthodontic treatment involving the bonding of fixed appliances to tooth surfaces can cause white spot lesions (WSLs). WSLs increase the likelihood of cavity formation and hence require preservation and prosthetic restoration. Therefore, the prevention of WSLs is of greater importance than treatment. Application of fluoride or the use of fluoride-containing mouthwash can prevent WSLs, but this requires patient cooperation and additional time and cost. Bioactive glass containing 2.5% fluoride was synthesized and mixed with the orthodontic bonding adhesive Transbond XT Low Flow (LV) at ratios of 1, 3, and 5% to prepare orthodontic adhesive samples. Scanning electron microscopy (SEM) and X-ray diffraction (XRD) were used to characterize the samples. The Vickers hardness test, bracket retention test, and adhesive remnant index (ARI) of the samples were analysed to determine their mechanical properties. To determine the biological cytotoxicity, the cell activity of the samples was evaluated using cell viability tests and the antibacterial activity was analysed using *Streptococcus mutans*. To evaluate the anti-demineralization effect, the sample was bonded to extracted teeth and a pH cycle test was performed. Micro computed tomography data were obtained from the bonded teeth and sample, and the anti-demineralization effect was evaluated using the ImageJ software program. The Vickers hardness of the sample was higher than that of LV and was dependent on the concentration of fluoride-containing bioactive glass (FBAG). The bracket retention test and ARI of the sample showed no significant differences from those of LV. The cell viability test showed no significant changes at 24 and 48 h after application of the sample. The fluoride ion release test indicated an ion release rate of 9.5–17.4 μg/cm^2^. The antibacterial activity of the experimental group containing FBAG was significantly higher than that of the LV group. The anti-demineralization test showed a concentration-dependent increase. However, the resin containing 5 mass% FBAG (FBAG5) showed a statistically-significant increase compared with LV. The orthodontic adhesive containing FBAG showed antibacterial and anti-demineralization effects, thus indicating possible WSL prevention activity.

## 1. Introduction

**Figure 1:**
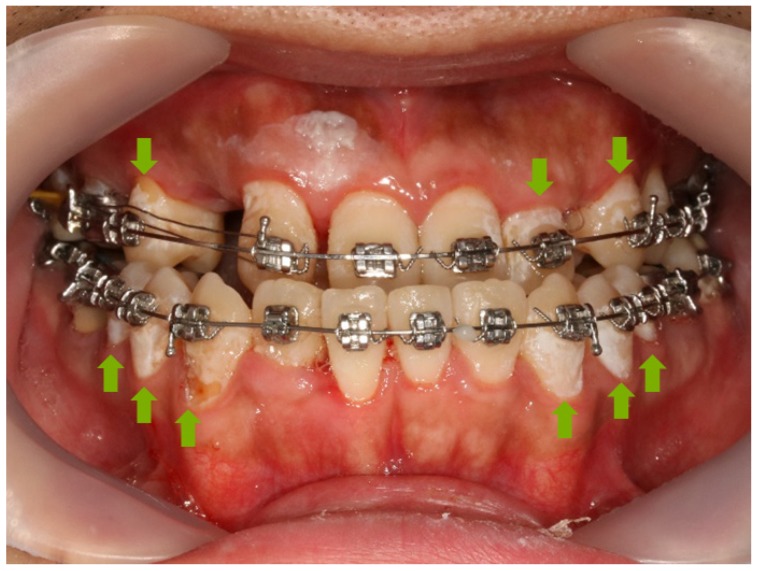
The WSL on orthodontic patient. Green arrow indicates WSL on tooth.

Demineralization is a serious complication associated with orthodontic treatment. A white spot lesion (WSL) manifests as an opacity of the affected tooth, and makes the tooth appear whiter than the unaffected teeth due to an increase in porosity under the enamel surface caused by carious demineralization [1,2]. The occurrence of WSLs in patients who received orthodontic treatment varies widely from 4.9% to 84% [3,4]. WSL occurs more frequently in treated maxillary incisors than in untreated maxillary incisors, a orthodontic treatment that adversely affects tooth appearance [5] (Figure 1). Plaque formation due to orthodontic appliances promotes the growth of *Streptococcus mutans* (*S. mutans*), which is caries-causing bacteria and a major cause of pH reduction [1,6,7]. Organic acids produced by bacteria decrease the pH to 5.5 or lower and WSLs are formed due to demineralization, mainly in the lower part of the loose band or in the periphery of the bracket base [3]. Another cause of WSLs in orthodontic patients is the demineralization resulting from an excessive loss of minerals from the enamel surface due to the application of the acid used to increase the bonding of orthodontic appliances. Excessive etching for bonding purposes increases the susceptibility of the enamel surface to demineralization and leads to iatrogenic WSLs [6]. The method of preventing WSLs in orthodontic patients is to apply fluoride. Fluoride intervenes in the metabolic processes in bacteria to inhibit or control their action and reduce the production of acids that can demineralize the surface of the teeth. Furthermore, fluoride forms fluorapatite, which increases the resistance of teeth to acids. The fluorapatite that fluoride makes on the tooth surface is more resistance for the acidic condition than for hydroxyapatite [8]. Although F^−^ is hydrophilic and cannot pass the bacterial lipid bilayer, HF is able to pass through the cell wall. The HF that passes through the cell wall breaks down into H^+^ and F^−^, which lowers the intracellular pH. The decreased intracellular pH causes changes in essential enzyme activity within the cell and kills the bacteria [8]. Fluoride can be applied to prevent demineralization of the teeth by adding it to toothpaste, using a high concentration fluoride varnish, or periodically using fluoride mouthwash. In addition, fluoride application is an intermittent process and requires additional time and financial costs because fluoride application for treatment can only be performed when patients visit a hospital. The use of fluoride toothpastes, mouthwash, and varnishes is fully dependent on patient cooperation; therefore, its effects cannot be relied on for uncooperative patients and children in particular. One method that does not require patient cooperation is the addition of anti-demineralization biocompatible materials, such as fluoride, to the bonding system. Although glass ionomer cements containing fluoride have been used as orthodontic adhesives, their bracket retention force is relatively low compared with that of conventional resin bonding [9]. Furthermore, studies on existing commercial fluoride-releasing adhesives and glass ionomers have revealed that they do not demonstrate a significant advantage in terms of their anti-demineralization effects compared with products that do not release fluoride [10]. To overcome these drawbacks, resin-modified glass-ionomer cements (RMGICs) have become the subject of various studies. However, orthodontists are more familiar with acid-etching techniques. Another available bonding system additive is bioactive glass (BAG), which is added to adhesives or resins [11,12]. The advantages of BAG include its action as a filler that does not reduce the bracket retention force [11]. BAG is a bioactive compound composed of CaO, Na_2_O, and P_2_O_5_ with a SiO_2_ base. The ions of the BAG is released and saturated around BAG. When the BAG was on the tooth surface, saturated ions from the BAG convert the amorphous calcium phosphate layer into apatite and crystallizes [13,14]. BAG also releases ions, which increase the pH and act as a buffer. In other words, BAG decreases the likelihood of developing WSLs near orthodontic brackets through its antibacterial effect and also prevents demineralization due to its action in increasing pH [15]. Remineralization effects can also be expected from the addition of the fluoride in BAG because it leads to the formation of apatite [16]. The objective of this study is to synthesize a fluoride-containing BAG and create an orthodontic bonding agent containing the BAG. In addition, the study includes an evaluation of the clinical applications of this orthodontic bonding agent through the conduction of a mechanical test and an evaluation of its antibacterial and remineralization properties.

## 2. Materials and Methods 

### 2.1. Synthesis of Fluoride-Containing Bioactive Glass (FBAG)

The BAG and FBAG were synthesized in accordance with the sol-gel method as follows [17,18]: 23 mmol of tetraethyl orthosilicate (TEOS, Product Number: 131903, Sigma-Aldrich, St. Louis, MO, USA) was added to 24 mL of ethanol. The pH of this solution was adjusted to 1–2 through the addition of 1 mol/L HNO_3_ (Samchun, Seoul, Korea). Subsequently, 14 mmol and 12.77 mmol of Ca(OH)_2_ (Sigma-Aldrich, St. Louis, MO, USA) were added to synthesize the BAG and FBAG, respectively. Then, 11.5 mmol of NaOH was added to each solution. In addition, 1.23 mmol of NaF (Sigma-Aldrich, St. Louis, MO, USA) was added to continue the synthesis of the FBAG. Thereafter, 1.25 mmol of (NH4)_2_HPO_4_ was dissolved in 400 mL of distilled water and added to both solutions to complete the synthesis of the BAG and FBAG. The pH of each solution was then set to 11 using an ammonia solution. Once the pH was adjusted, distilled water was added until the final volume was 600 mL. Each solution was then stirred for 48 h, dried at 60 °C for 24 h, and finally heated to 600 °C in a furnace for 6 h. 

### 2.2. Characterization of FBAG

The physical structure of the samples was analysed using field-emission scanning electron microscopy (FESEM, S-4700, Hitachi, Tokyo, Japan). The X-ray diffraction (XRD) patterns of BAG and FBAG were analysed using an automated X-ray powder diffractometer (Ultima 4, Rigaku, Tokyo, Japan) with Cu Kα radiation (λ= 1.5409292 Å) at 40 kV and 40 mA. The step size was set at 0.020°, and scanning rate at 1.50°/s in the 2θ range from 10° to 50°. The typical functional groups of BAG and FBAG were evaluated at wavelengths of 400–4000 cm^−1^ using FT-IR (Spectrum GX, PerkinElmer, MA, USA) and the kernel ridge regression (KRR) method. 

### 2.3. Preparation of a Resin Disk Containing FBAG

Disks (diameter 5 mm, height 2 mm) were fabricated to evaluate the properties of the orthodontic bonding adhesives containing FBAG. The 30 resin disks were prepared for Vickers hardness, bracket retention test, antibacterial test and cell viability assay. To prevent light transmission, 2 mL of orthodontic bonding adhesive LV (Transbond Supreme LV Low Viscosity Light Cure Adhesive, 3M, Monrovia, CA, USA) was added to three black 1.5 mL Polypropylene conical microcentrifuge tube (HD4323K, Vernon Hills, IL, USA), with FBAG concentrations of 1, 3, and 5 mass%, respectively. They were then stirred twice for 10 s each using a mixer (TORNADO SHM-ALM00, Shinhung, Seoul, Korea). The homogenized samples were inserted into brass molds, pressed with a slide glass (t: 0.2 mm), and light-cured for 20 s using light curing unit (VALO; Ultradent Products, South Jordan, UT, USA). The samples containing FBAG concentrations of 1, 2, and 5 mass% are hereafter referred to as FBAG1, FBAG2, and FBAG5, respectively.

### 2.4. Vickers Hardness

The Vickers hardness was measured using a microhardness tester (MVK-H1, Mitutoyo, Kanagawa, Japan) by applying a loading force of 200 gf to the top of the prepared resin disk. Three samples were used for each group, and each sample was measured three times. 

### 2.5. Bracket Retention Test

To evaluate the bracket bonding strength of the synthesized bonding agents, the bracket retention force was measured using a universal testing machine (Instron Corporation, Canton, MA, USA). Five premolars extracted during orthodontic treatment were allocated to each group. The premolars used in the test did not possess any WSLs or enamel defects. The surface on which the bracket was to be bonded was rinsed with fluoride-free pumice using prophylaxis cups, flushed for 10 s, and then dried. The premolars were etched for 15 s using a 35% phosphoric acid gel (Ultra Etch, Ultradent, South Jordan, UT, USA), suctioned, flushed, and dried. A light-cured adhesive primer (Transbond XT; 3M, Nonrovia, CA, USA) was applied to the dry premolar surface and air was blown gently over the surface for 2 s. The sample was bonded to the bracket (Orthos AP Metal Bracket, Ormco, CA, USA) parallel to the long axis of the tooth. The residual paste was removed, and the mesial and distal parts were light-cured for 5 s. This entire process was performed in accordance with the manufacturer’s recommendations for the use of Transbond XT Primer. The bracket-bonded teeth were immersed in distilled water for 24 h and then measured using a universal testing machine (Instron Corporation, Canton, MA, USA). The machine’s steel rod indenter (width: 1 mm) was placed perpendicular to the bracket, and the maximum load (N) was measured at a crosshead speed of 1 mm/min. The measured load (N) value was divided by the bracket base area (12.98 mm^2^) and converted to bond strength (MPa). The bonding failure of the debonded tooth surface was evaluated using an adhesive remnant index (ARI) score (Table 1)

### 2.6. Antibacterial Properties

The disks were placed in 96-well plates and bonded to the bottom surface of the plates using the same light-curing resin used for the control group. The 96-well plates were disinfected using a low-temperature plasma (LOWTEM Crystal 50, LowTem Co., Gunpo-si, Korea) before being used in the experiment. *S. mutans* with a concentration of 1.0 × 10^5^ CFU/mL was placed in a brain heart infusion medium (Becton Dickinson, Cat. No.: 237500, Franklin Lakes, NJ, USA) and cultured in an incubator at 37 °C. The absorbance was measured at a wavelength of 620 nm after culturing for a period of 24 h and again after a period of 48 h (Sunrise, TECAN, Männedorf, Switzerland).

### 2.7. Cell Viability Assay

The cytotoxicity of the orthodontic bonding agents containing FBAG was evaluated using MTT-assay. Disks were placed in the 96-well plates and disinfected using a low-temperature plasma (LOWTEM Crystal 50, LowTem Co., Gunpo-si, Korea) before being used in the experiment. Human gingival fibroblasts (HGF-1, ATCC, Manassas, VA, USA) were cultured in Dulbecco’s modified Eagle’s medium (DMEM, Hyclone, Logan, UT) containing 10% fetal bovine serum (FBS, Hyclone, Logan, UT) and 100 IU/mL penicillin/streptomycin (Hyclone, Logan, UT). The HGF-1 cells were injected into the 96-well plates containing the samples and were cultured for 24 h and 48 h in a CO_2_ incubator at 37 °C. After culturing, MTT (3-(4,5-dimethylthiazol-2-yl)-2,5-diphenyltetrazolium bromide) (Sigma-Aldrich, St. Louis, MO, USA) at 5 mg/mL concentration was added and reacted for 4 h in a dark room. Thereafter, the supernatant was removed and MTT crystals were dissolved in 150 μL dimethyl sulfoxide (DMSO, Sigma-Aldrich, St. Louis, MO, USA) formed in the cells, and the absorbance at 620 nm was measured using a microplate reader (Sunrise, TECAN, Männedorf, Switzerland). 

### 2.8. In Vitro F Dissolution Test

To evaluate the F ion release capacity of a sterile resin disk, ion dissolution (ICS-5000, ThermoFisher, Dionex, MA, USA) was performed. The sterile resin disk and 5 mL of simulated body fluid (SBF, Biosesang, Seongnam-si, Korea) were inserted into a 5 mL tube, and the ion concentrations released from the resin disk were measured after a period of 0.5, 1, 5, 10, and 20 days. The SBF is consisted with 0.71 M Na^+^, 25 mM K^+^, 12.7 mM Ca^2+^, 7.7 mM Mg^2+^, 0.7397 M Cl^−^, 21 mM HCO_3_^−^, 5 mM HPO_4_^2−^, 2.5 mM SO_4_^2−^ and pH is 6.7

### 2.9. Remineralization Properties

**Figure 2:**
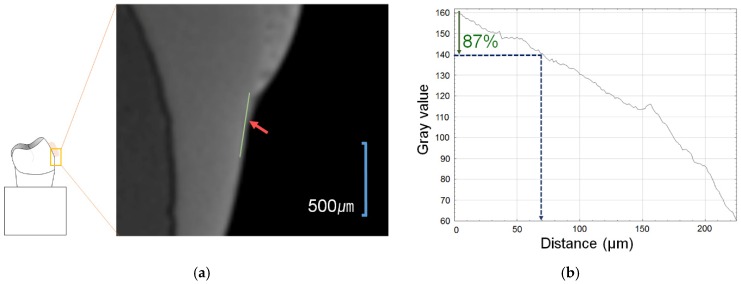
Remineralization length analysis method. (**a**) Microcomputer tomography CT slice of the region of interest (ROI) at the center of the lesion perpendicular to the enamel surface. The starting point was the end of the adhesive, the green line is the line separating the ROI from a reference point on the enamel surface. (**b**) Histogram from the ImageJ software program. The blue arrow indicates up to a level of 87% gray value from the reference point, the red arrow indicates the distance to the 87% gray value from the reference point.

The remineralization properties of the orthodontic bonding agents containing BAG were evaluated using the pH cycling protocol. This protocol has been shown to make artificial caries like lesions. [19]. The teeth used in this study were premolars extracted during orthodontic treatment and possessed no WSLs or other enamel defects. Each group was allocated five premolars. The pH cycle for the remineralization evaluation was as follows: 

Each tooth was buried in acrylic resin (Caulk Orthodontic Resin, Dentsply Caulk, York, PA, USA) using a mold. The surface of the buried tooth sample to be bonded was rinsed with fluoride-free pumice and a prophylaxis cup, flushed for 10 s, and dried. To prevent etching except within the target 5 mm × 5 mm tooth surface, the vertices of the 5 mm × 5 mm square surface were clearly marked with nail varnish. The 5 mm × 5 mm tooth surface was etched for 30 s with 35% phosphoric acid gel (Ultra-etch, Ultradent, South Jordan, UT, USA), flushed for 10 s, and dried. The orthodontic bonding adhesive sample, which was manufactured using the same procedure as used for the disks, was applied to the surface and was light-cured for 5 s. The teeth were then immersed in distilled water for 24 h. The remineralization solution and demineralization solution were ordered to solution company by customized as Table 2. The samples were immersed in 500 mL of demineralizing solution (Biosesang, Seongnam-si, Korea) for 6 h, then washed distilled water 1 min, and finally immersed in 500 mL of the remineralizing solution (Biosesang, Seongnam-si, Korea) for 18 h. This immersion cycle was repeated for a period of 14 days. The solutions were replaced afresh once a week. The samples were washed with distilled water for 1 min and dried with gently-moving air before replacing the demineralizing and remineralizing solutions. The samples were measured using a micro-CT (InspeXio SMX-90CT Plus Benchtop Micro Focus X-ray, Shimadzu, Japan) at 90 kV and 109 μA. The measured micro-CT data were analysed using the ImageJ software program (National Institutes of Health, Bethesda, MD, USA) [20] (Figure 2). The scale was calibrated by the original micro-CT scale bar. The starting point was the end point of the sample orthodontic bonding resin. Using ImageJ, the length was adjusted in accordance with the scale bar on the micro-CT. Sound enamel was defined as comprising 87% gray value using a histogram, and the remineralization length was determined by measuring the distance from the end point of the sample orthodontic bonding resin to a point beyond the 87% gray value. 

### 2.10. Statistical Analysis

The Shapiro–Wilk test was performed for a test of normality. Levene’s test was tested to assess the equality of variances. Vickers hardness and FBAG concentration were analysed by regression analysis. The inter-group difference (bracket retention test, cell viability test, antibacterial test) was statistically analysed by one-way analysis of variance (ANOVA) test followed by Duncan’s post-hoc test. Kruskal-Wallis test was performed to analyse the nonparametric ARI. The anti-demineralization analysis of no-normal distribution was performed by Kruskal-Wallis. After Kruskal-Wallis, the difference means between the two groups were tested by the Mann-Whitney test. The significance level was set to 5%. Every statistical analysis was performed with R language program (Version 3.6.0; R Foundation for Statistical Computing, Vienna, Austria).

### 2.11. Ethics Statement

The bracket retention test and remineralization property test were approved by the IRB (Institutional Review Board of Pusan National University Dental Hospital (PNUDH-2018046)). 

## 3. Results

### 3.1. Characterization

**Figure 3:**
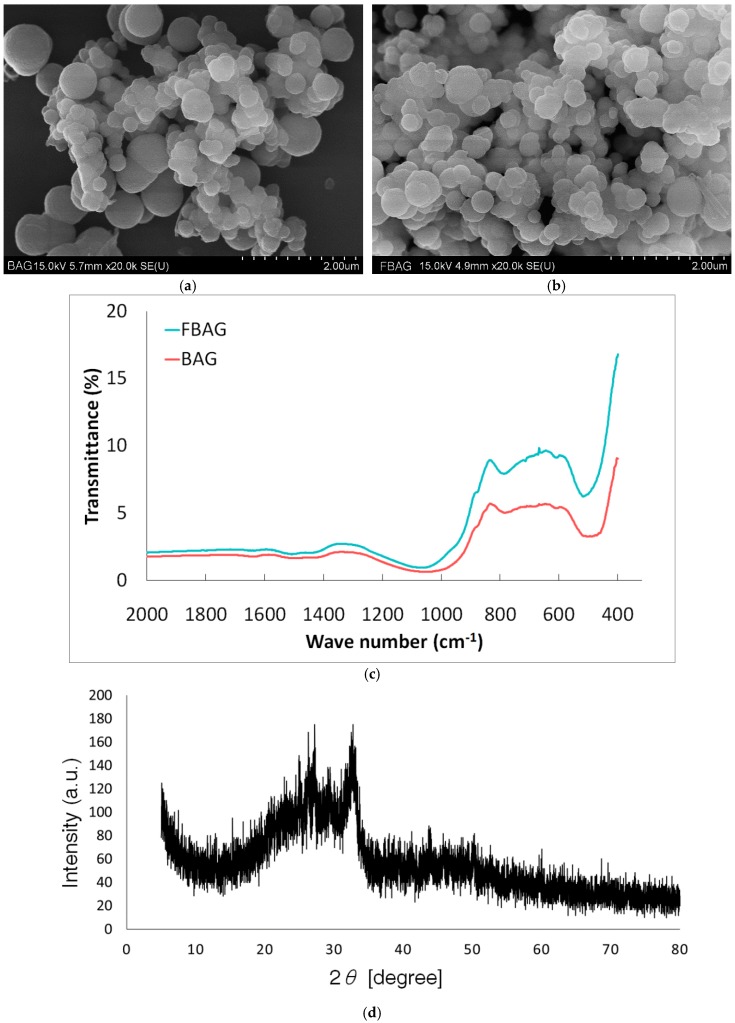
BAG and FBAG characterization. (**a**) SEM image of BAG; (**b**) SEM image of FBAG; (**c**) FTIR spectra of BAG and FBAG; (**d**) XRD patterns of BAG.

SEM showed that the BAG had irregular morphology and consisted of a large number of nanoparticles. Figure 3 shows that the BAG and FBAG synthesized using the sol-gel method in this study exhibited particle aggregation [21]. In the FTIR spectra, BAG and FBAG silicate bands for the Si–O–Si bending mode were observed at 540–470 cm^−1^ [21]. The XRD pattern did not show evidence of a crystalline peak as reported by a previous study [12]. 

### 3.2. Vickers Hardness

**Figure 4:**
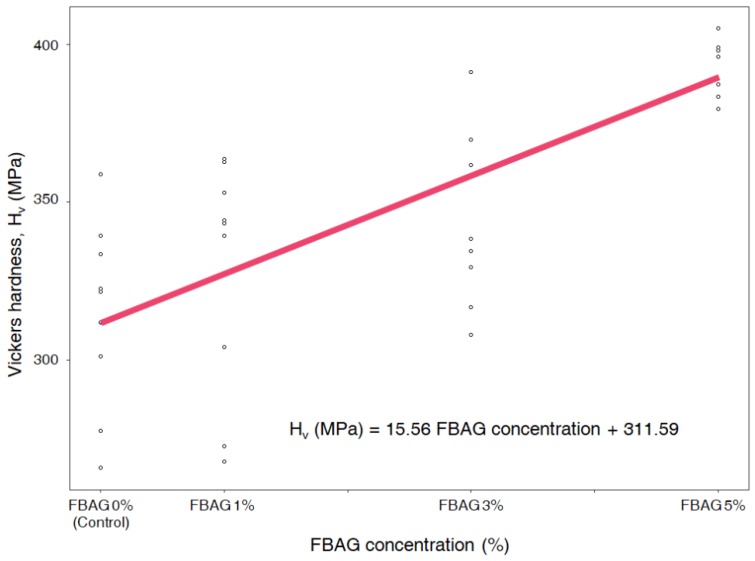
Correlation between concentration of FBAG and Vickers hardness in resin disk (r = 0.54; *p* < 0.001).

Vickers hardness of FBAG in resin disk was positively correlated with the concentration of BAG (r = 0.54; *p* < 0.001) (Figure 4). Vickers harness of LV group was 314.8 ± 9.8 MPa, FBAG1 was 327.6 ± 12.8 MPa, FBAG3 was 349.1 ± 9.8 MPa and FBAG5 was 359.2 ± 2.9 MPa (Figure 4).

### 3.3. Bracket Retention Test

**Figure 5:**
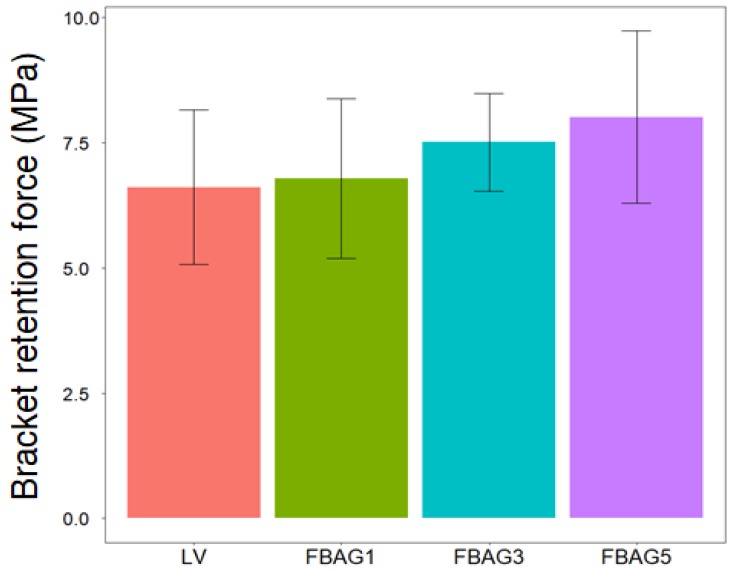
Bracket retention test of LV and FBAG containing group. There was no difference between the groups by one-way ANOVA (*p* > 0.01). Error bars represent ± standard error (n = 5).

The LV (6.6 ± 1.5 MPa) showed no statistically-significant differences in comparison with FBAG1 (6.8 ± 1.6 MPa), FBAG3 (7.1 ± 0.6 MPa), and the FBAG5 (8.0 ± 0.9 MPa) (*p* > 0.05) (Figure 5).

### 3.4. Adhesive Remnant Index (ARI) Score

**Figure 6:**
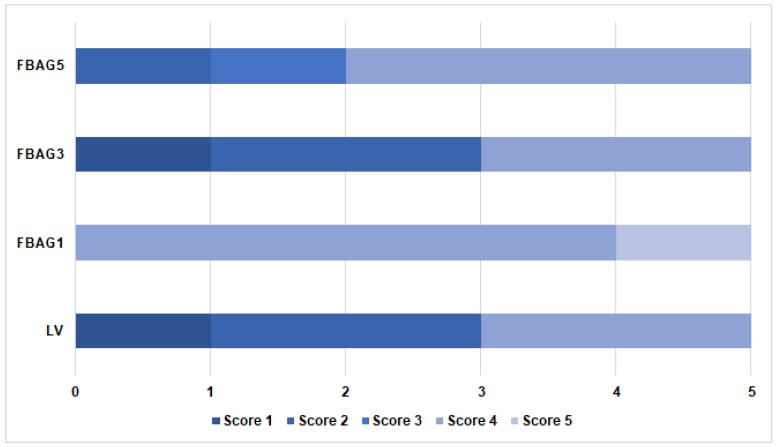
ARI scores for evaluated orthodontic bonding adhesives. The ARI score was not significantly different according to the Kruskal-Wallis test at α = 0.05 (n = 5).

A comparison of the ARI scores for the LV group (2.6 ± 1.3), FBAG1 (4.2 ± 0.4), FBAG3 (2.6 ± 1.3), and FBAG5 (2.4 ± 0.9) did not demonstrate statistically-significant differences, as can be seen in Figure 6.

### 3.5. Cell Viability Test

**Figure 7:**
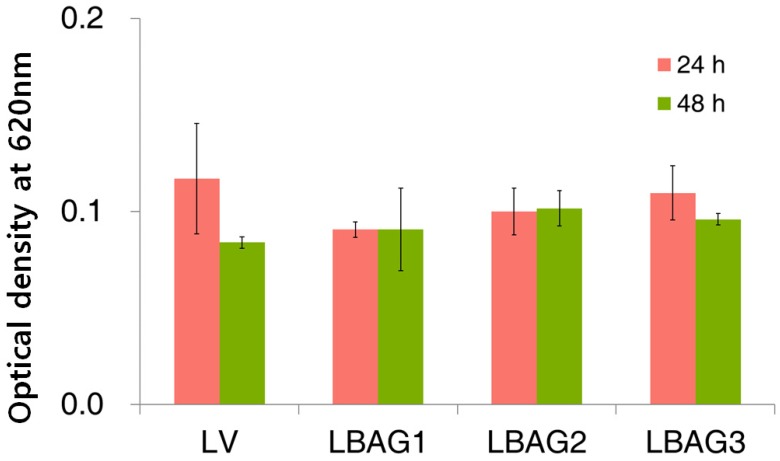
Cell viability results (after 24 and 48 h) using HGF-1 cytotoxicity on LV and FBAG-resin disk. The one-way ANOVA was used to compare the cell viabilities within groups for 24 h and 48 h (*p* > 0.05). Error bars represent ± standard error.

After 24 h, the optical density for FBAG1 (0.09 ± 0.00), FBAG3 (0.10 ± 0.01), and FBAG5 (0.11 ± 0.01) decreased compared with the LV group (0.12 ± 0.03); however, the differences were not statistically-significant. After 48 h, there were no statistically-significant differences among the cell viabilities; the optical density of FBAG1 (0.09 ± 0.02), FBAG3 (0.10 ± 0.01), and FBAG5 (0.10 ± 0.00) were not significantly different with the LV group (0.08 ± 0.00) (Figure 7). 

### 3.6. Antibacterial Test

**Figure 8:**
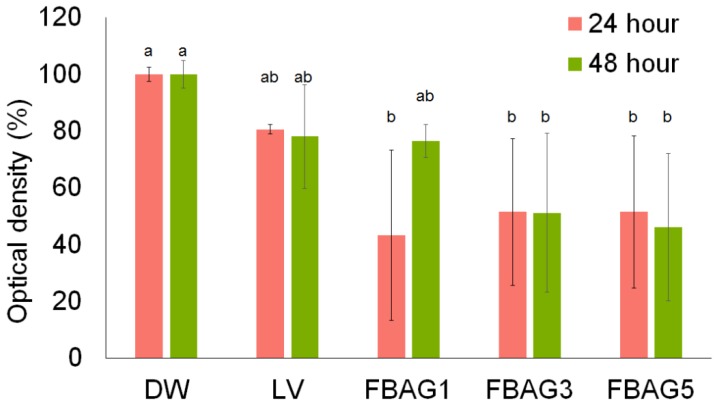
The difference in antibacterial properties between cured LV and FBAG orthodontic bonding pastes at 24 and 48 h. The same letter indicates no statistically significant difference between the groups (*p* < 0.05) by Duncan’s multiple comparison test. Error bars are shown ± standard error.

Untreated blank was used as a reference with an optical density of 100%. The antibacterial properties after 24 h for FBAG1 (43.4 ± 21.2%), FBAG3 (51.5 ± 14.9%), and FBAG5 (51.5 ± 15.5%) were significantly higher than those of the LV group (80.6 ± 1.4%) (*p* > 0.001). Furthermore, the antibacterial properties after 48 h for FBAG1 (76.4 ± 3.4%), FBAG3 (51.2 ± 16.2%), and FBAG5 (46.2 ± 14.9%) were higher than those of the LV group (78.1 ± 10.5%) (*p* > 0.001), as shown in Figure 8. Compared with distilled water (DW) and LV, the group containing FBAG showed a significant antibacterial effect.

### 3.7. In Vitro F Dissolution Test

**Figure 9:**
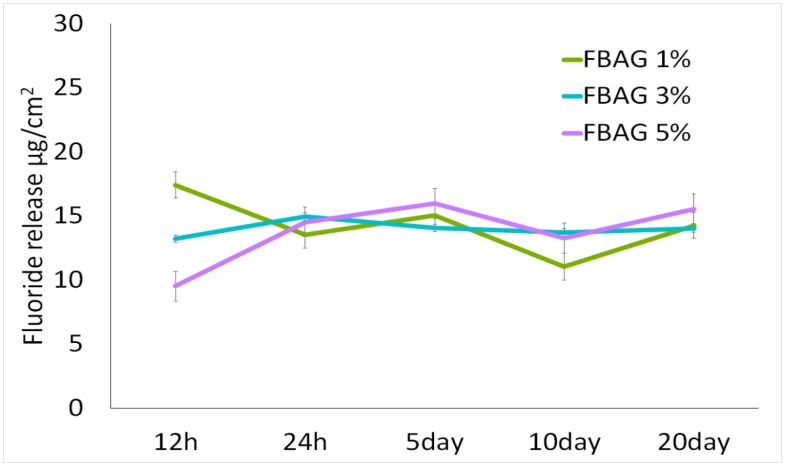
Fluoride release from FBAG1, FBAG3, and FBAG5. Resin disks of diameter 5 mm and thickness 2 mm were prepared and used.

After a period between 0.5 and 20 days, the fluoride ion release was 11.1–17.4 μg/cm^2^ for FBAG1, 13.2–14.91 μg/cm^2^ for FBAG3, and 9.5–15.6 μg/cm^2^ for FBAG5 (Figure 9).

### 3.8. Anti-demineralization

**Figure 10:**
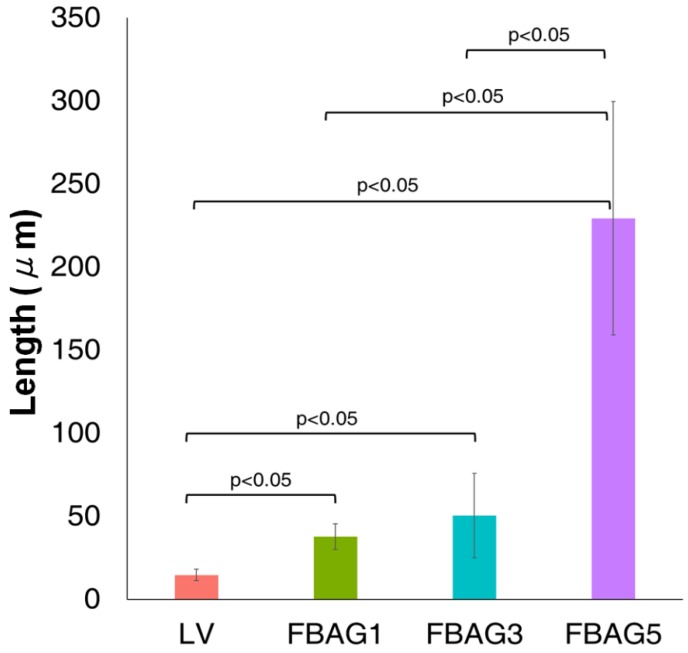
Anti-demineralization length comparison of orthodontic bonding adhesive containing LV and FBAG using ImageJ analysis. The Kruskal-Wallis test was used to compare the shear bond strength within groups (*p* < 0.05). Mann-Whitney test was used to analyse the difference of means between groups. Error bars represent ± standard error.

**Figure 11:**
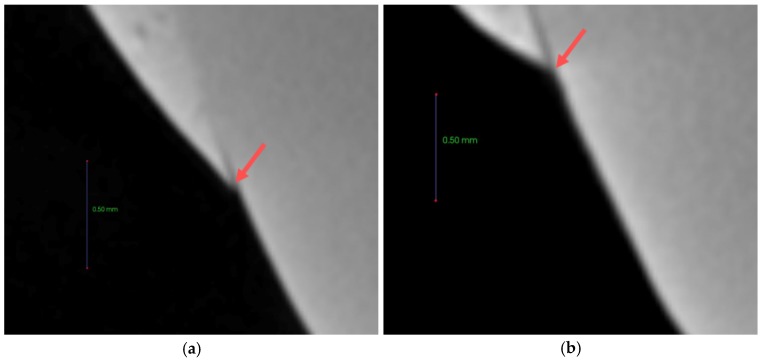

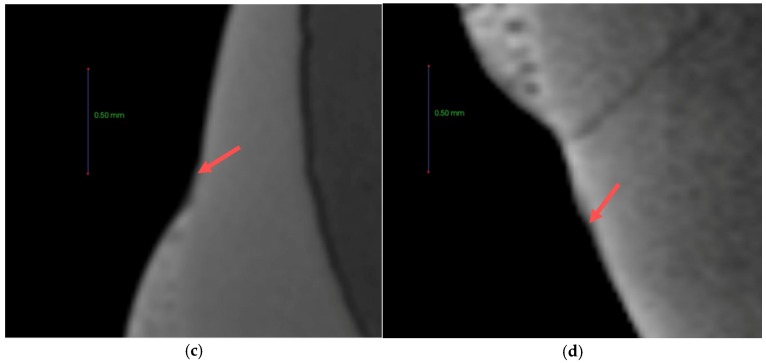
87% gray value point from the reference point of the LV and FBAG orthodontic bonding adhesive via micro-CT. (**a**) LV; (**b**) FBAG1; (**c**) FBAG3; (**d**) FBAG5.

The anti-demineralization results (Figure 10 and Figure 11) showed that the anti-demineralization distances of FBAG1 (37.9 ± 7.7 μm), FBAG3 (50.4 ± 25.3 μm), and FBAG5 (229.3 ± 70.2 μm) were significantly larger than those of LV (14.7 ± 3.4 μm). Compared with LV, the group containing FBAG showed a significant different.

## 4. Discussion

Many studies have been performed on the addition of biomaterials to orthodontic adhesives to prevent WSLs without any additional time or cost [11,12]. In this study, FBAG was added to orthodontic adhesives, and the possibility of its use in WSL prevention was investigated. 

The mechanical properties of the new orthodontic adhesives showed that the Vickers hardness was more concentration-dependent in the FBAG-containing group than in the LV group. Kim et al. and Lee also reported that adding BAG increased the Vickers hardness [11]. As with previous studies, no significant difference between bracket retention test and ARI was observed [11,12,22]. This suggests that adding up to 5% FBAG would cause no clinical problems with respect to the fixing of the orthodontic appliance. 

The biological evaluation results showed that no significant differences in cell viability were observed at 24 and 48 h compared with LV. Toxicity assay using gingival fibroblast-1 cells did not show any toxicity compared to LV already used in patients.

The ion exchange of H^+^ or H_3_O^+^ among network-modifier ions, such as Na^+^, K^+^, and Ca^2+^, released from the BAG [20]. Furthermore, the sodium, silica, calcium, and phosphate ions released from the surface of the BAG increased the salt concentration. The BAG decreased the proliferation of microorganisms via three mechanisms [22,23]. Another characteristic of fluoride that resulted in WSL prevention was its antibacterial activity. F^−^ is hydrophilic and cannot pass through the bacterial lipid bilayer, but HF can pass through the cell wall. The HF that passes through the cell wall breaks into F^−^ and H^+^, which decrease the intracellular pH, and can cause changes in the essential intracellular enzyme activity, which kills bacteria [8]. 

Similar to previous studies, the present study also showed that the antibacterial activity of the FBAG-containing group was higher than that of both the DW and LV groups, 24 and 48 h after application. 

The chemical remineralization effect of the pH cycle showed that as the FBAG concentration increased, the anti-demineralization effect increased in comparison to the LV. FBAG appeared to behave as a useful material that released more ions at lower pH [24]. Ion release not only had an antibacterial effect, but also lowered the pH. The low-pH environment that can lead to demineralization of the enamel surface was changed to a high-pH alkaline environment. Fluoride is also known to have a remineralization property [1]. Furthermore, over time, FBAG generates not only hydroxyapatite, but also fluoroapatite, which possesses superior hardness [16]. Similar to previous studies, a concentration-dependent anti-demineralization effect was noted in this study. As a result, it was shown that there was a possibility of remineralization in the demineralized area around the orthodontic appliance.

In the present study, orthodontic adhesives containing BAG demonstrated physical and biological stability, thereby enabling their use in clinical settings. Compared with previous control mechanisms, orthodontic adhesives containing BAG possessed superior antibacterial and anti-demineralization properties. Therefore, these results showed the clinical feasibility of supplementing mineral loss caused by etching for orthodontic appliance adhesion with BAG [6]. In addition, FBAG antimicrobial activity has a positive effect to prevent demineralization caused by lactic acid produced by plaque bacteria around the orthodontic appliance. Compared to bioactive glass with fluorinated graphite (FGtBAG), which was our previous study [25], FBAG is more suitable for clinical use than FGtBAG, because bracket retention force was not decreased. The reason was that FBAG was white, compared to FGtBAG, which was gray [25]. However, in this study, the antibacterial test was performed only with *S. mutans*. Hence, there is scope for future work to employ diverse bacterial species and pathways. Further in-vivo studies and studies using real clinical scenarios will be necessary. Although the orthodontic treatment period is a long term treatment for at least one year, this study design was a short term study. Ion-release from orthodontic adhesive containing ions decreased in 2–3 months [26]. In future studies, it will be necessary to establish a patient application protocol to demonstrate long-term effects. The filler of conventional dental resin composite is made of glass material. Because the filler represents various properties according to distributions, particle size, and shape [27], a variety of extended studies will be needed for better bonding ability and remineralization properties in further study.

## 5. Conclusions

FBAG demonstrated physical and biological stability sufficient for its clinical use in bonding resins. Anti-bacterial activity show that FBAG have a higher concentration-dependent anti-bacterial activity than LV. FBAG demonstrated an excellent anti-demineralization effect than LV. Therefore, orthodontic resins containing FBAG demonstrated the capacity to prevent WSLs. 

## Figures and Tables

**Table 1:** ARI score criteria.

Score	Evaluation Criteria (Remaining Adhesive on the Tooth)
1	All
2	More than 90%
3	Between 10–90%
4	Less than 10%
5	No adhesive

**Table 2:** Composition of demineralizing and remineralizing solution.

The Solutions	Composition
Demineralizing solution (pH 4.4, 0.5 L)	Ca(NO_3_)_2_∙4H_2_O 2.0 mM
	KH_2_PO_4_ 2.0 mM
	CH_3_COOH 75.0 mM
Remineralizing solution (pH 7.0, 0.5 L)	Ca(NO_3_)_2_∙4H_2_O 1.5 mM
	KH_2_PO_4_ 0.9 mM
	KCl 130 mM
	NaC_2_H_6_AsO_2_∙3H_2_O 20.2 mM
